# Advancing understanding of microbial bioenergy conversion processes by activity-based protein profiling

**DOI:** 10.1186/s13068-015-0343-7

**Published:** 2015-09-25

**Authors:** Yun Liu, James K. Fredrickson, Natalie C. Sadler, Premchendar Nandhikonda, Richard D. Smith, Aaron T. Wright

**Affiliations:** Beijing Key Laboratory of Bioprocess, College of Life Science and Technology, Beijing University of Chemical Technology, 100029 Beijing, China; Biological Sciences Division, Pacific Northwest National Laboratory, 902 Battelle Blvd, MSIN J4-02, Box 999, Richland, WA 99352 USA

**Keywords:** Activity-based protein profiling (ABPP), Cellulosic bioethanol, Biodiesel, Protein redox, Proteomics

## Abstract

The development of renewable biofuels is a global priority, but success will require novel technologies that greatly improve our understanding of microbial systems biology. An approach with great promise in enabling functional characterization of microbes is activity-based protein profiling (ABPP), which employs chemical probes to directly measure enzyme function in discrete enzyme classes in vivo and/or in vitro, thereby facilitating the rapid discovery of new biocatalysts and enabling much improved biofuel production platforms. We review general design strategies in ABPP, and highlight recent advances that are or could be pivotal to biofuels processes including applications of ABPP to cellulosic bioethanol, biodiesel, and phototrophic production of hydrocarbons. We also examine the key challenges and opportunities of ABPP in renewable biofuels research. The integration of ABPP with molecular and systems biology approaches will shed new insight on the catalytic and regulatory mechanisms of functional enzymes and their synergistic effects in the field of biofuels production.

## Background

The need for sustainable and renewable energy alternatives to replace fossil fuels has become a global issue that requires highly innovative research and development [1]. Strategies to identify new organisms and enzymes with desired biofuel-relevant capabilities or alternatively, genetically modifying model organisms for optimized productivity, will require improved annotations and mechanistic insights into functional cellular processes, including transcriptional regulation, post-translational protein modifications and clues into regulatory networks [2]. Conventional genomic, transcriptomic, and proteomic measurements provide broad insight into the architecture of a biological system, but largely fail to accurately assess the functional and regulatory details of microbial cells. Therefore, the post-genomic era requires new, complementary approaches to characterize biochemical pathways of microorganisms representing the vast untapped biodiversity of microbial ‘dark matter’ and associated uncharacterized and undiscovered functional enzymes and pathways [3, 4]. Achieving such strategies will augment our understanding of the microbial metabolism required for efficient and economical production of biofuels and specialty chemicals. An emerging and powerful approach is activity-based protein profiling (ABPP), a chemical biology probe-based technology that can reveal details of microbial catalytic and regulatory functions for bioenergy-relevant applications.

An ABPP strategy fills critical knowledge gaps that cannot be determined from genome technologies that only detect and quantify the abundance of macromolecules (RNA, protein, metabolites) [5–7]; namely, the status and identity of active enzymes, metabolite/substrate-protein interactions, and enzyme regulation. The significant feature of ABPP is the selective identification of the functionally active proteoform of a target enzyme using chemical probes, which react with an active site residue of a particular enzyme (or enzyme family) in a mechanism-based fashion. Additionally, probes can be used to selectively identify protein regulatory modifications (e.g., Cys thiol redox) [8] and substrate/small molecule-protein interactions [9, 10]. Thus, ABPP has the ability to characterize the function of unknown proteins, improve gene annotations, and provide measurement of alteration to individual enzyme activities under differing conditions or perturbations. The ABPP capability may also facilitate the discovery of new biofuel pathways by confirming and discovering putative enzymes and their roles in metabolism and regulation. In doing so, new organisms with ideal functionality may be revealed, or insights into targeted genetic recombination strategies for optimized biofuel production may be designed based on ABPP results.

To date, several excellent reviews or book chapters have described small-molecule probes designed to profile diverse classes of enzymes and the bio-orthogonal chemistry and ABPP applications in chemical biology research, drug screening, and target proteins related to disease pathology [5–7, 11, 12]. However, there is limited information on ABPP technology in bioenergy development. In this review, we focus on the development of ABPP strategies for improved understanding and design of microbial systems for bioenergy production, including cellulosic bioethanol, biodiesel and phototrophic production of hydrocarbons.

### Fundamental elements of the ABPP technology

Emerging as a key technology in the evolution of functional proteomics, the root of ABPP can be traced back to the development of radioisotope and fluorescent probes that were used to track metabolic activities and subcellular disposition of small molecules, respectively. The rapid growth of high resolution and high sensitivity LC–MS-based proteomics brought about the evolution of ABPP, where probe readout enables identification of specific protein targets, their quantification and even subcellular locale. ABPP has also been coupled with fluorescence microscopy and other techniques enabling targeted imaging of subcellular function and localization [13–15]. Since the initial profiling of serine proteases by Cravatt’s group in 1999 [16], various ABPs have recently been developed to target diverse enzyme classes, such as serine hydrolases [7, 17], cysteine proteases [17, 18], metalloproteases [19], kinases [20], ATPases and nucleotide-binding proteins [21], deubiquitylases [12], cytochrome P450s [22], histone deacetylases [23, 24], glutathione-S-transferases [25] and glycosidases [26–29].

**Fig. 1:**
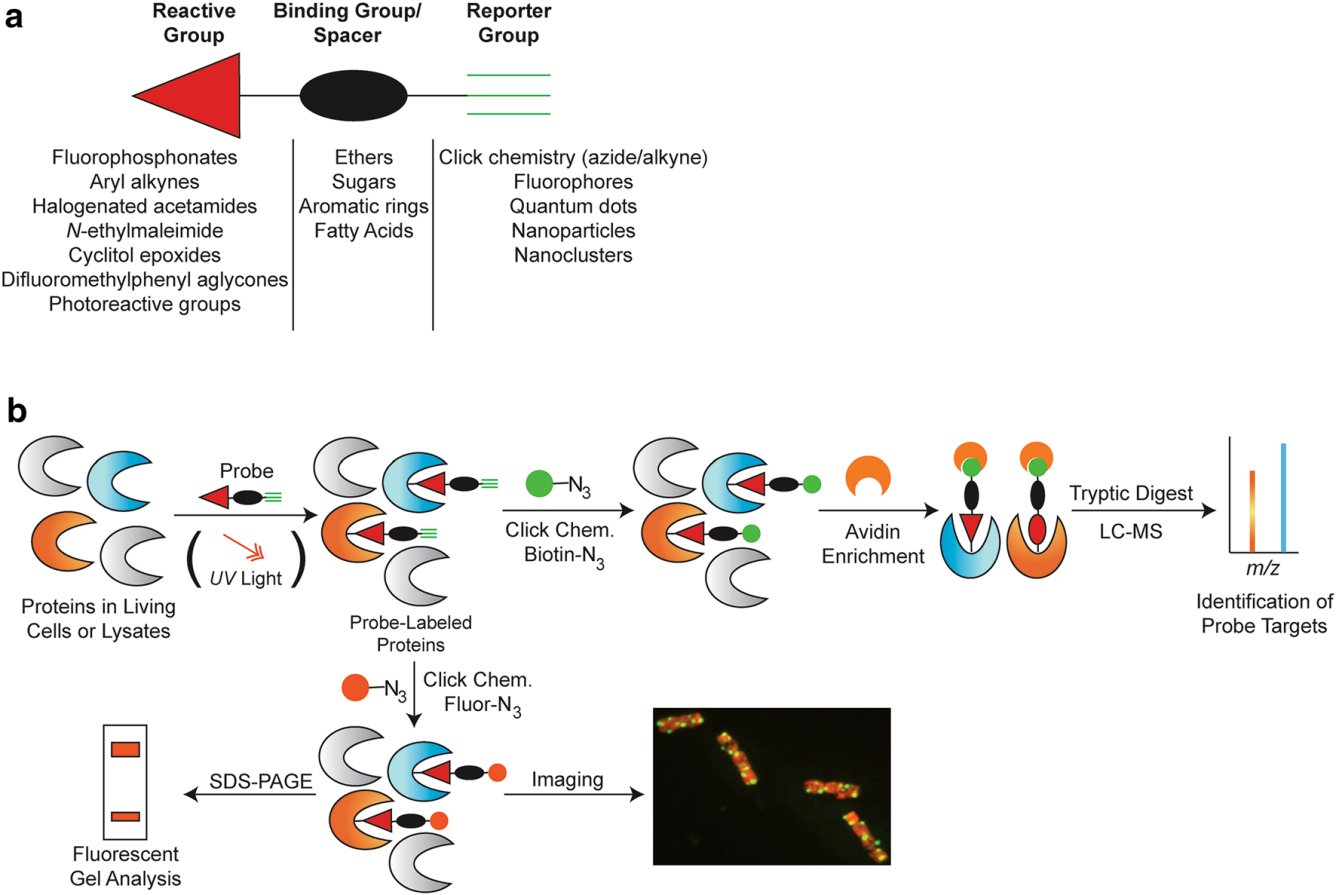
Multimodal measurements enabled by ABPP. **a** Generic format of an activity-based probe, including a reactive group for irreversible binding to an enzyme target, a binding group that biases the probe toward a particular class of enzymes and/or a spacer region that can impart cell permeability, and a reporter group to enable multimodal readout of probe labeling. **b** The activity-based probes are added directly to living cells or cell lysates and irreversibly bind target proteins. Click chemistry enables the addition of biotin for enrichment and quantitative liquid chromatography–mass spectrometry (LC–MS) of probe targets. Alternatively, fluorophores are added by click chemistry for gel analysis of target proteins, imaging to quantify uptake and distribution of labeling, and/or cell sorting to quantify uptake

Activity-based protein profiling relies on ABPs that form stable covalent bonds with active proteins in complex proteomes by direct reaction with protein residues or via photoreactive crosslinking. The ABPs consist of three moieties: (1) a reactive group that forms an irreversible covalent bond with a target enzyme or protein. Sulfonate esters, fluorophosphonates, peptide acyloxymethyl ketones, aryl alkynes, iodoacetamides, and cyclitol epoxides are examples of reactive groups that have been successfully incorporated into ABPs; (2) a binding group (“substrate mimic”) that biases the probes toward an enzyme family and may also impart cell permeability, and (3) a reporter tag for rapid and sensitive purification and characterization of labeled enzymes (Fig. 1).

To facilitate ABPP studies directly within the native physiological context of proteomes under study, the use of bio-orthogonal reactions (“click chemistry”) enables the use of reporter tag-free probes in which probe labeling is performed prior to reporter conjugation [30, 31]. Reporter tags (e.g., fluorophore or biotin) can be directly attached to the alkyne or azide group on the probe after it has bound its target via copper-catalyzed or copper-free bio-orthogonal click chemistry reaction. This allows the probe size to be small, thereby minimizing undesirable impacts on reactivity with target proteins, and maximizing transport and cell permeability. Furthermore, it permits the facile exchange of the type of reporter that is applied to probed targets based on the desired application and outcome of the study, and properties of the sample being assayed (Fig. 1). Most common is the addition of biotin as a reporter to facilitate resin-based enrichment of ABP-targeted proteins for subsequent proteomic measurements, and fluorescent dyes for cell imaging or fluorescence-activated cell sorting. The advent and deployment of bio-orthogonal labeling strategies has greatly broadened the analytics applied to ABP-labeled systems, and has enabled important in situ analyses.

### ABPP in cellulosic bioethanol production platforms

#### Glycoside hydrolases (GHs) in cellulose biohydrolysis

The rapid improvement of biofuel production from renewable lignocellulosic biomass has been made a high priority due to the growing worldwide energy demand and the threat of global warming associated with the combustion of fossil fuels. Biologically derived energy (‘bioenergy’)-relevant enzymatic catalysis plays a critical role in biomass conversion [2]. Enzymatic lignocellulose hydrolysis is a key process in terrestrial carbon cycling and constitutes a critical function supporting the growth of soil microbial heterotrophs. It is important in plant–microbe interactions and holds significant promise for conversion of lignocellulosic biomass to valuable carbon-based products. Lignocellulose is a complex, heterogeneous biopolymer, and hence its enzymatic hydrolysis requires numerous glycoside hydrolases (GHs) from various enzyme families, including cellulases, hemicellulases, pectinolytic enzymes, lignin catabolizing enzymes, and cell wall loosening enzymes. More than 130 GH families have been identified [32], of which at least 40 are involved in cellulose hydrolysis for bioethanol development, and these enzymes with well-coordinated synergy can achieve high cellulose hydrolysis efficiency [33, 34]. Therefore, characterizing specific individual enzyme activities and the synergistic interactions of these cellulases is crucial for optimizing cellulose bioethanol development, but is extremely difficult to obtain due to heterogeneity within biological systems. Traditional enzyme assays, such as colorimetric cellulase and xylanase activity assays, enzyme immunosorbent assays, gel electrophoresis, chromatographic and capillary electrophoresis based separations only provide readout of the total mixture activity towards a defined substrate, rather than individual enzymes. However, a GH activity-based probe (GH-ABP) can directly identify and quantify functionally active cellulolytic enzymes in native secreted proteomes, allowing high-throughput analysis in vivo and/or in vitro via gel electrophoresis or LC–MS-based proteomics [28, 29, 35]. This approach can also identify synergistic interactions between GH enzymes [36]. Looking forward it is anticipated that ABPP will play a critical role in facilitating the whole processing of lignocellulosic bioethanol production by microbial enzymes.

#### GH-probe classification and target enzymes

Since 1992, chemical probes designed to target GHs enzymes have been developed, and these probes have achieved a remarkable success in monitoring GHs activities [26–29, 36–43]. Early probes were not used as ABPs, but rather to understand enzyme structure, active site substrate preferences, and catalytic mechanisms. Activity-based probes have opened the door to specific functional characterization of individual enzymes within complex proteomes and under native physiological conditions. Table 1 summarizes GH-probe structures and their enzyme targets that have been used for proteomic and imaging applications.

**Table 1:** GH-ABPs used to characterize functional activity in microbes relevant to bioethanol production; categorized by either unbiased screens or targeted inhibitor GH-ABP strategies

Labeling mechanism	GH-ABP structure	Target enzyme	References
Unbiased screening		α-Hexosaminidase	[37]
		Peptide-*N*-glycosidase	[72]
		β-Glycosidases	[38]
		α-Glycosidases α-Galactosidases α-Mannosidases	[39]
		β-1,4-Glycanase	[26]
		β-Glycosidases	[40]
		Cellulase	[28, 41]
		β-Glycosidases	[42]
		α-Galactosidase	[73]
		Glycosidase I	[29]
		Glycoceramidase	[43]
		Cellulase	[28]
		Exo-glycosidases	[35]
Small molecule inhibitor		Acid β-Glycosidases	[29]
		β-Glycosidases	[28, 29]
		Acid β-Glycosidases	[74]
		β-Glycosidases	[28, 29]
		β-Glycosidases	[28, 29]
		β-Glycosidases	[74]

**Fig. 2:**
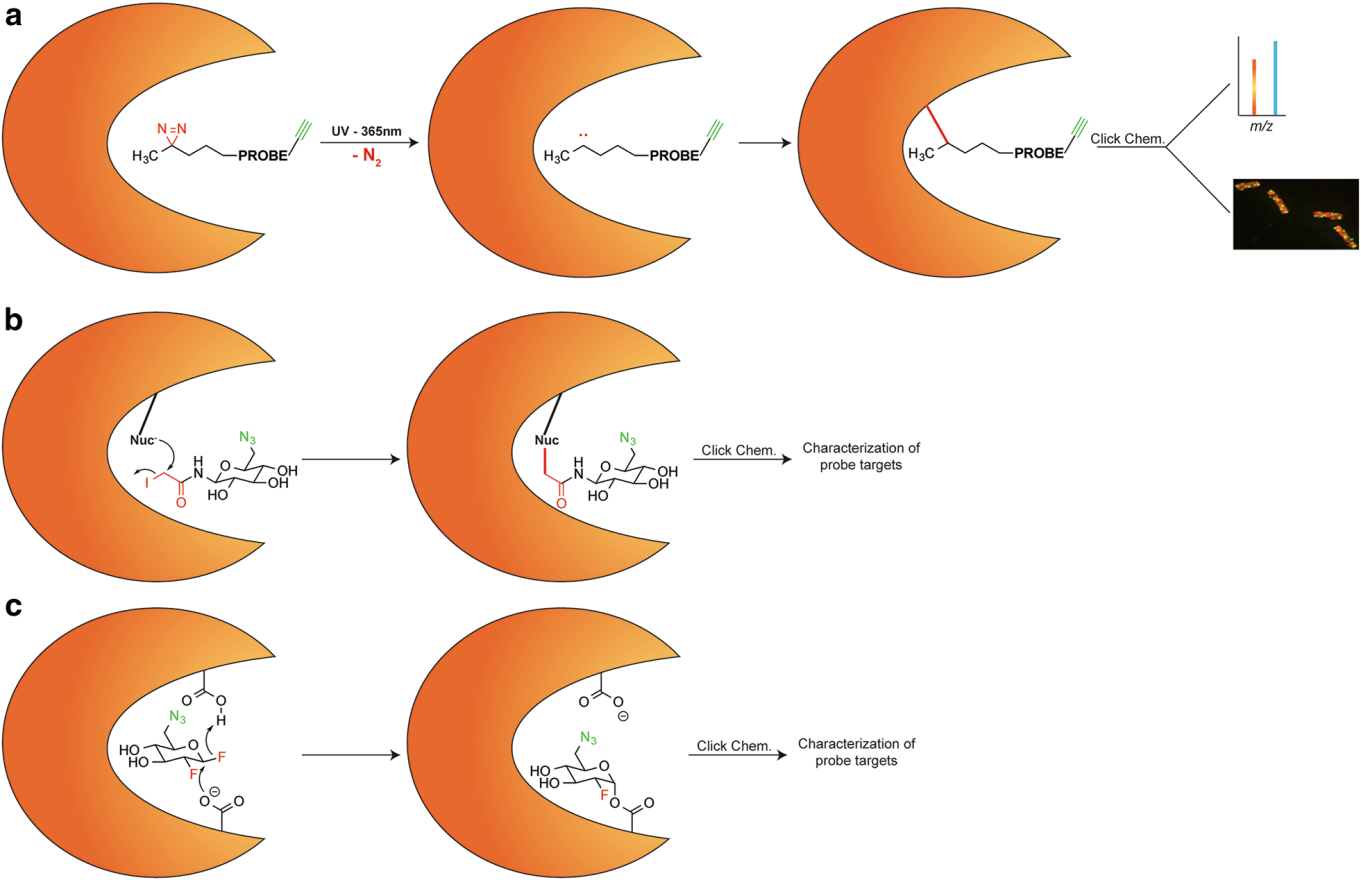
Representative mechanisms for labeling of glycoside hydrolase enzymes by a variety of probe types. In each image the *orange shape* represents a target enzyme. **a** Photoreactive diazirine-functionalized probes work by probe intercalation into the substrate binding site, followed by UV irradiation and formation of a carbene that performs C–H insertion into the peptide of the target protein. Subsequent click chemistry is used for characterization of probe targets, e.g., by imaging or mass spectrometry. **b** Following association of the probe into the substrate binding region of the enzyme, the electrophilic iodoacetamide moiety of the probe reacts with a nucleophilic amino acid residue of the enzyme in a classic nucleophilic displacement reaction. **c** Mechanism-based probes work by directly interrogating the catalytic machinery of the enzymes, resulting in a covalent complex between probe and enzyme

The probes shown in Table 1 employ a variety of molecular mechanisms that result in covalently labeled target enzymes (Fig. 2). This includes photoreactive mechanisms, such as those probes with diazirines, aryl azides, or benzophenone. These probes are added to lysates or a secreted protein fraction, incubated for a period of time, then UV irradiated to form covalent bonds (Fig. 2a). A second approach has been the use of halogenated acetamides, such as chloro- or iodoacetamide, which are attached at the anomeric position of a sugar. These probes interact with carbohydrate metabolizing enzymes, and label a reactive, nucleophilic amino acid within or near the enzyme active site due to their electrophilicity (Fig. 2b). The final group of probes, such as the fluorosugars and cyclitol epoxides take direct advantage of the catalytic mechanism of the target enzyme families, functioning as mechanism-based inhibitors. For instance, the vast majority of β-glycosidases use a catalytic mechanism employing two carboxylate residues, one that acts as a nucleophile and a second that performs as a general acid/base catalyst (Fig. 2c). To target these enzymes the probe irreversibly traps the glycosyl-enzyme intermediate. Due to the fact that many ABPP applications are intentionally broad, meaning profiling of a class of enzymes is desired rather than a single isoform, off-targets are an issue, and appropriate controls and statistics must be performed to limit false discoveries. Off-targets can be mitigated by careful tuning of the concentration of probe applied, and by wise selection of controls such as competitive inhibition and no probe added samples.

Glycoside hydrolase probes have been applied to a wide swath of applications ranging from identifying novel and known protein activities in both sequenced and un-sequenced genomes, and determination of the subcellular localization of probe reactive enzymes [5, 35]. A key challenge for developing this class of probes lies in the point of attachment of the reporter group. For instance, exo-glycosidases have a pocket-shaped active site in which extensive contacts are formed between the protein and all substrate hydroxyl groups, making it impractical to incorporate a bulky biochemical reporter group such as biotin. An example of a successful solution to this problem was reported by Stubbs and co-workers who developed an improved strategy by designing tag-free variants of a probe and phosphine-FLAG and alkyne-biotin as post labeling reporter tags that successfully enabled the probe profiling of exo-glycosidases in *Pseudomonas aeruginosa* [42]. Numerous other reports (see Table 1) have also used a two-step click chemistry-mediated labeling approach to circumvent the challenges of designing a bulky synthetic probe capable of binding in the active site of GH enzymes akin to native ligands; these probes incorporate either an azide or alkyne for the click chemistry reaction. To characterize glycosidases that do not proceed through a traditional covalent glycosyl-enzyme intermediate photoreactive ABPs have been employed, incorporating diazirines, benzophenones, or aryl azides [43]. The complexity in designing robust and selective GH probes was exemplified in an important manuscript comparing the efficiency of labeling with fluorosugar- or cyclitol epoxide-based probes, and also the use of direct labeling (e.g., probe attached to reporter) versus click chemistry-enabled labeling strategies. Witte and co-workers demonstrated that endogenously expressed GH levels were most efficiently labeled by the epoxide probes, and that a two-step click chemistry labeling approach can be employed, but careful probe design must be made or the two-step approach is poor [29]. The insights from this manuscript have been important to subsequent probe developments and applications.

#### GH-ABPs applied to cellulose-degrading microbes

Chemical probe profiling of GHs has proven to be an efficient tool for profiling GH enzyme activities and their synergistic effects in vitro, in situ, and in vivo [27, 44]. Thermophilic microbes are highly attractive candidates for conversion of lignocellulose to bioethanol because: (1) they produce robust and efficient carbohydrate degrading enzymes; (2) they can survive the harsh lignocellulose pretreatment conditions employed industrially to expose polymeric sugars from lignin; and (3) the harsh thermochemical treatments are reflective of their natural biotopes. Chauvigné-Hines and co-workers used a suite of GH-ABPs (difluoromethylphenyl aglycone, 2-deoxy-2-fluoroglycoside, and *N*-halogenated glycosylacetamide) to directly label the cell-adherent cellulosome of the model anaerobic thermophilic organism *Clostridium thermocellum* [28]. Employing a proteomic analysis, the authors used the GH-ABPs to reveal the dynamic assembly and reactivity of the *C. thermocellum* cellulosome grown on microcrystalline cellulose. Importantly, a diverse array of type I dockerin-containing GH enzyme families and additional accessory protein functions were characterized. A total of 177 GH enzymes, including 84 carbohydrate active proteins, 41 dockerin-containing proteins and 52 GH family proteins were quantitatively identified by the ABPs in *C. thermocellum*. An interesting aspect of this manuscript is the off-target labeling incurred as a result of the reactivity of two of the probe types employed, the difluoromethylphenyl aglycone and *N*-halogenated glycosylacetamides. The primary targets of these probes are the β-glycosidases, however the reactivity of the *N*-halogenated glycosylacetamides resulted in labeling of other protein types that also interact with sugars (e.g., glycosyl transferases). Difluoromethylphenyl aglycones label indirectly as a result of the catalytic mechanism of GHs, in which the enzyme-probe reaction results in a transient electrophile that labels a nucleophile within the active site, but the electrophile can also diffuse away and label neighboring proteins, particularly those in complexes. Thereby, other cellulosomal proteins were identified. In this report, the off-target labeling was controlled by careful consideration of the probe concentration, but off-target labeling was also beneficial by providing a broader window into sugar catabolism by *C. thermocellum*. As indicated earlier, careful experimental design and controls must be deployed to enable appropriate biological conclusions.

Anderson et al. [36] used *N*-iodoacetylglycosylamine derived probes from the GH-probe suite synthesized by Chauvigné-Hines et al. [28] to comprehensively compare, identify, and quantify cellulolytic enzymes activities in *Trichoderma reesei* wild-type strain QM6a and mutant strains NG14 and RUT-C30. *T. reesei* is both a model fungus for cellulose degradation and an industrial platform. Mutant strains have been developed to optimize cellulase production and limit catabolite repression in commercial bioethanol production platforms. A total of 45 proteins were characterized as a function of culture age, and 32 proteins showed significant shifts among the different strains as a function of changing time. Importantly, alterations to enzyme function due to growth variables (pH, temperature, salts, etc.) could be rapidly identified, including both subtle and dramatic changes. This is broadly applicable to characterizing existing and newly identified microbes with useful biomass degrading properties. GH-ABPs have high potential for identifying new enzymes, synergistic functions, regulatory modifications, and other critical protein activities to enhance and optimize enzyme cocktails for lignocellulosic biomass processing.

The vast majority of activity-based probes for the GH enzyme family are designed from substrate mimics. However, most research reports have not systematically characterized the selectivity and binding efficiencies of the probes versus natural substrates. Competition experiments are often performed, but the natural substrate is used in considerable excess versus the probe. As described earlier, this is in part a result of the desire to broadly profile enzyme classes, rather than single enzymes. But, this also imparts higher rates of off-targets. To ameliorate this chemists will need to use computational ligand docking approaches and/or synthetic production of large numbers of probes for subsequent screening of highly selective probes. With regard to biofuels, research has been primarily focused on broadly characterizing cellulose deconstruction mechanisms applied by various microbes, and highly selective probes for single enzymes have not been needed.

The development of GH-ABPs will facilitate a more comprehensive understanding of enzyme function for bioethanol platforms by targeting specific enzymes, producing insight into the formation and catalytic dynamics of cellulosome and free enzyme systems. Additionally, the probe-based method provides rapid experimental feedback to characterize the activities and shifts in activities of GHs. It is likely that the method will be adopted for high-throughput screening capabilities via gel electrophoresis or LC–MS-based proteomics to produce highly efficient cellulase cocktails for industrial bioethanol production from lignocellulosic biomass.

### Opportunities for ABPP in biodiesel production

#### ABPP of lipase function for biodiesel production from oil lipids

Biodiesel is an alternative fuel source in which first-generation biodiesels have been synthesized from edible oils [45], and second-generation biofuels are procured from non-edible and microbial oils [46–48]. Biodiesel has become a sustainable, non-toxic, biodegradable fuel substitute that can be employed in the current diesel transportation infrastructure [49]. Compared with conventional chemical-catalyzed biodiesel production, enzymatic catalysis (e.g., lipases) has been regarded as a highly promising processing technology [50]. Appropriately, a large number of assays have been developed to analyze their activity in vitro [51], including chromogenic assays, quantitation of released fatty acids, fluorescent substrates assays, and ABPP. The use of ABPP tied to ELISA-based assay is capable of measuring active-site engagement of triglyceride lipase family proteins in complex mixtures [52]. Though ABPP has not been applied yet to the delineation of active enzymes directly involved in catalyzing biodiesel production, analysis of the prior reports on novel probe designs for characterizing and quantifying lipase function, primarily in human disease systems, has revealed that there is great promise for using ABPP for characterizing enzyme functions applicable to biodiesel development [53–57].

Tam and co-workers reported a prototypic lipase-specific ABP, biotinylated M352, and an ABP-based ELISA assay to quantitatively measure endothelial lipase activity in cells, which could potentially be used for high-throughput measurements of lipase activity [52]. The authors demonstrated that ABPs could be employed to measure other members of the triacylglycerol lipase family, such as lipoprotein lipase. Prior lipase-ABP designs will be highly applicable to probes employed for biodiesel production. The structure and polarity of the ABP can have a large impact on probe target specificity and can be tuned towards lipolytic enzymes by including hydrophobic elements in the structure. Table 2 describes ABPs relevant to targeting lipase enzymes previously described in the literature.

**Table 2:** Activity-based protein profiling of lipase enzymes

No	Lipase-ABP structures	Tag	Target lipase	Refs
1		Biotin- TAMRA-	Serine hydrolases	[75]
2		Biotin- Fluorescein- Rhodamine-	Serine hydrolases	[16, 76]
3		Biotin- Fluorescein- Rhodamine-	Serine hydrolases	[77]
4		NBD-	Serine hydrolases	[54]
5		NBD- Biotin- Biotin-S-S-	Serine hydrolases	[78]
6		NBD- Biotin- Biotin-S-S-	Lipases	[78]
7		NBD- Biotin- Biotin-S-S-	Cholesterol esterases	[78]
8		Biotin-S-S-	Lipases	[53]
9		Biotin-S-S-	Lipases	[53]
10			Fatty acid acyl carrier protein (ACP), thioesterase (TE)	[59]
11				[59]
12				[59]
13		Biotin- Rhodamine-	Fatty acid synthase enzyme (FASE)	[79]
14			Monoacylglycerol lipase (MAGL)	[60]
15			Lipases	[57]
16			Lipases	[58]

#### ABPP of fatty acid metabolic enzyme for biodiesel production

Microbial oils or fuel precursors, like those produced by yeast growth on biomass-derived sugars and algae through photosynthesis, are ideal feedstock sources for biodiesel as some of these organisms are genetically tractable and can be cultivated for high yielding oil production [58]. However, without a detailed understanding of spatiotemporal enzyme activity and regulation of fatty acid production, engineering microbes to meet growing energy demands will be a considerable challenge. Suitable modulation of fatty acid synthase enzyme activity could enhance the oils level in microbial growth. An exciting future prospect is the use of the ABPP technology for the identification of differentially active enzymes in fatty acid metabolism pathways. Additionally, ABPP may help identify regulatory limitations upon metabolite production and may facilitate the engineering of microbial strains optimized to maximize metabolite secretion, thereby improving overall economics of biofuels production.

Regulation of enzymes involved in lipid metabolism is governed by a complex network of protein–protein and protein–small molecule interactions. ABPP technology can be used to monitor the key lipolytic enzymes functions related to oil lipid metabolism (Table 2). For instance, Blatti and co-workers employed an activity-based crosslinking probe (chloroacylic pantetheine probe) to selectively trap transient protein–protein interactions between the fatty acid acyl carrier protein (ACP) and the thioesterase (TE) governing fatty acid hydrolysis within the algae *Chlamydomonas reinhardtii* [59]. The authors demonstrated in vitro that this species’ TE must functionally interact with ACP to release fatty acids. These findings highlight the critical role of protein–protein interactions in manipulating fatty acid biosynthesis for algae biofuel engineering as illuminated by activity-based probes. Attention to lipase enzyme regulation by ABPP could be used to increase our understanding of the fatty acid metabolic pathways leading to elevated oil accumulation in microbial cells. Another ABPP example for spatiotemporal profiling was performed by Chang and co-workers who used hexafluoroisopropyl carbamate probes to create in situ imaging profiles of monoacylglycerol lipase (MAGL) and α-β hydrolase-6 (ABHD6), which are directly related to fatty acid metabolism [60]. Finally, Viertler and co-workers used a hexynyl-4-nitrophenyl-hexylphosphonate fatty acid probe to image the subcellular localization of active lipases [57]. Therefore, the use of ABPP to profile the activity of fatty acid-associated enzymes in living cells should provide new opportunities to understanding oil lipid metabolism of biodiesel feedstocks in the near future. We anticipate that this is feasible in current model organisms, but ABPP may also be applicable to the characterization of new organisms with useful metabolic characteristics.

### ABPP of reduction–oxidation sensitive proteins in phototrophs

Current approaches to genetically modifying phototrophs to generate high titers of the molecules of interest, e.g., liquid alkanes, lipids, and hydrogen, have run into considerable issues because the underlying regulatory processes governing the allocation of carbon and reductant into specific biomass components or desired metabolic products are poorly understood. Post-transcriptional modifications of proteins have been shown to be important for the temporal regulation of photosynthesis and other fundamental metabolic processes in microbes [5, 8, 61, 62]. Protein reduction and oxidation (redox) reactions comprise one type of highly efficient post-translational modification that cells use to regulate metabolic activity in diverse ways. However, protein redox events are elusive and difficult to capture due to their labile and oxygen sensitive nature [63, 64]; cell lysis can disrupt the native redox context of the cell, significantly perturbing or destroying sensitive modifications and thereby producing non-biologically relevant artifacts. Therefore, reducing and alkylating agents or acid capture techniques have been required to try and maintain physiologically relevant redox states upon cell lysis, but most recently in vivo ABPP has been employed [64].

Several reports have reported light-dependent disulfide/dithiol exchange of redox regulation of phototrophs [61, 62, 65]. More than 100 targets of thioredoxin and glutaredoxin have been identified in *Arabidopsis*, spinach, poplar, and multiple cyanobacteria and algae species to date [62, 64]. Zhu and co-workers identified 65 and 118 potential redox-responsive proteins in *Brassica napus* guard cells treated with abscisic acid and methyl jasmonate, respectively, by gel electrophoresis and isotope-coded affinity tagging methods [66]. However, these reports could only identify potential redox-susceptible proteins because the studies were performed on cell lysates.

**Fig. 3:**
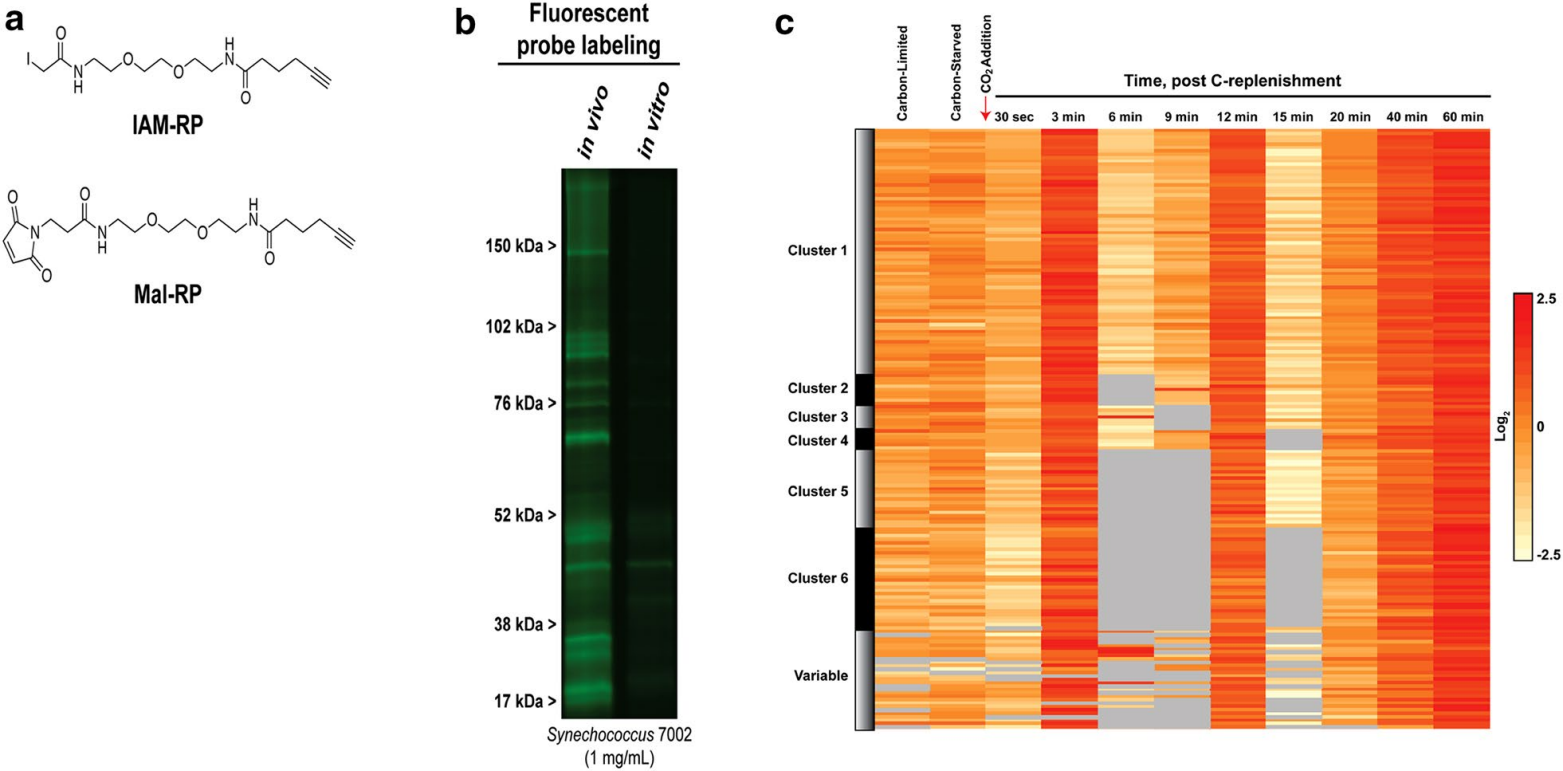
ABPP developments for live cell profiling of protein cysteine thiol redox dynamics. **a** ABP structures derived from iodoacetamide (IAM) and *N*-ethylmaleimide (Mal) electrophiles known to react with reduced thiols. **b** A fluorescent gel of in vitro and in vivo IAM-RP labeling of the cyanobacterium *Synechococcus* 7002. Oxidation due to lysis clearly results in significant aberrations to the protein redox status of the cell; **c** *Synechococcus* 7002 cells were grown in a photobioreactor and available carbon was limited. The cells were then starved of carbon followed by addition of CO_2_. Protein redox dynamics were profiled before and after the addition of carbon for 60 min. The heat map of in vivo IAM-RP and Mal-RP-labeled proteins shows large temporal dynamics in probe labeling. The heat map portrays times when specific proteins are most reduced (*red*) versus more oxidized (*light yellow*). *Gray coloring* indicates that oxidation is so significant that no detectable probe labeling was observed. Reprinted with permission from [64]. Copyright 2014 American Chemical Society

Recently, Sadler and co-workers applied iodoacetamide and *N*-ethylmaleimide derived electrophilic activity-based redox probes designed with the capability of cell permeability to cyanobacteria (Fig. 3) [64]. The probes were applied to the photoautotrophic cyanobacterium, *Synechococcus* sp. PCC 7002, both in vitro and in vivo to compare redox-sensitive protein dynamics. The results demonstrated more effective labeling of redox-sensitive proteins in vivo than in vitro due to lysis-induced oxidation. The authors also performed experimental shifts with C availability by inducing C-starvation followed by C-replenishment over a 60 min time span, thereby inducing a flux in intracellular reductant partitioning. Of the 176 probe-labeled proteins, 101 were newly identified as redox-sensitive, while the remaining 75 had been previously found as regulated by Trx or susceptible to redox in cyanobacteria (*Synechocystis* 6803) or plants (*Arabidopsis thaliana*).

Later, Ansong, Sadler and co-workers employed IAM-RP and Mal-RP to characterize redox-sensitive protein dynamics in live *Synechococcus* sp. PCC 7002 cells during light-to-dark transitions and carbon and nitrogen limitation [67]. The authors used quantitative proteomics to demonstrate that probe-labeled redox-sensitive proteins showed significant difference between light and dark conditions (i.e., sensitive to light-to-dark transition). Strikingly, the abundance of all 300 identified redox-sensitive proteins by LC–MS increased in the dark relative to the light condition indicating widespread protein reduction as result of the light-to-dark transition. Additionally, numbers of labeled redox proteins under carbon- and nitrogen-limited conditions were higher compared to the nutrient replete cultures, indicative of a more reduced cellular environment. These findings were consistent with a lack of terminal electron acceptors (N rich nucleotides and CO_2_), which results in the over-reduction of plastoquinone and NADP^+^.

Collectively, these results provide insight pertaining to the roles protein redox reactions play in reacting to environmental perturbations such as nutrient limitation and diel cycling. A clear understanding of the feedback loops, signaling events, and PTMs used by cells to modify function is essential for gaining a higher resolution of metabolic processes and modulation. Hydrocarbon biosynthesis and H_2_ productions by phototrophs are exciting alternative fuel sources, but both processes are sensitive to and may require specific redox interactions for activation [68, 69]. By identifying proteins involved in dynamic redox processes and the environmental conditions that perturb their balance, novel strategies can be applied to modify target organisms, or alternatively, optimize culture conditions for efficient production of target molecules.

In addition to redox-based regulation, ABPP also has potential for providing insights into other forms of post-translational regulation in cyanobacteria and other microorganisms relevant to biofuels production. For example, protein phosphorylation regulates a range of critical functions in cyanobacteria including carbon and nitrogen metabolism, photosynthesis, and stress response but identifying targets of signaling pathways is a major challenge [70].

## Conclusions

There is considerable promise for microbial bioenergy conversion. The field of ABPP is poised to yield new insights into the enzymes responsible for biosynthetic and catabolic processes, as well as the regulatory mechanisms cells utilize for partitioning carbon and energy.

To more fully realize the benefits of ABPP for bioenergy production, several challenges must be overcome. This includes the selection of important new probe targets, synthesis of novel probes which are capable of targeting and exploring new classes of enzymes and their poorly understood catalytic mechanisms, tight substrate specificity, and/or low expression levels in biofuels development. Simultaneously, the application of ABPP will need to shift from probe labeling studies in well defined axenic microbial cultures, and expanded into characterizing the enzymes dynamics in mixed microbial cultures and complex environmental or engineered microbial communities. The engineering of designer communities would benefit greatly from ABPP strategies. A conceivable approach would include the identification of novel microbes that demonstrate biofuel-relevant processes that could be coupled with photoautotrophic microbes to yield a self-sustaining culture. Additionally, it is likely that considerable realms of functional phylogeny are yet to be identified; ABPP may play an important role in function-enabled mining of microbial communities. Novel enzymes with non-canonical sequences and/or tolerance to extreme conditions may also be identified with high efficiencies for lignocellulose catabolism, lipases, transesterases, and other biofuel-relevant functions. ABPP can also be utilized to establish high-throughput SDS-PAGE based assays for screening and determining ideal culture conditions that induce high enzyme activity levels for biofuel-relevant metabolic processes. Finally, ABPP coupled with fluorescence and electron microscopy, Raman spectroscopy, and other techniques may elicit a much improved understanding of functional protein dynamics at the spatial and interaction scales. This knowledge could be used to create microbial communities for targeted biofuels production, or to understand and mitigate drivers of cyanobacteria or algae pond crashes. Regardless of the biofuel target, ABPP is currently being used to functionally characterize microbes and microbial communities, and future prospects for this application are limitless.

Over the past few years, ABPP has boomed in the field of chemical biology through elucidation of target functional proteins in vivo and in vitro. ABPP techniques that integrate transcriptomics, proteomics, fluorescence and electron microscopy, molecular biology (e.g., structural biology, mutant and gene cloning technologies), and other disciplines will provide comprehensive characterization of the catalytic and regulatory mechanisms of target enzymes, enzyme complexes, and entire biosynthetic pathways, thereby accelerating systems-level understanding required for efficient and economic biofuel technology development. Where transcriptomic and global proteomic analyses can only be used to infer functional capability in a biological system, ABPP directly measures function. There are exciting opportunities for coupling omic strategies to ABPP, such as linking post-translational modifications (e.g., phosphorylation, acetylation, redox) directly to enzyme function. Recent advances in the burgeoning field of intact proteomics may soon enable such comprehensive and high-throughput analyses that assign post-translational modifications to an enzyme’s active state. Additionally, lipidomic and metabolomics analyses may be coupled directly to ABPP data to correlate metabolic output directly to protein functions.

Beyond biofuels research and development, ABPP can fill a critical need for new technologies to connect uncharacterized microbial species or guilds to ecosystem processes. While metagenome sequencing has paved the road towards establishing these linkages, critical technical and bioinformatic challenges remain [71]. ABPP cannot only inform protein function, but can be directly assigned to genes via LC–MS-based proteomics. This provides a powerful approach for improving functional annotation of genomes including those of uncultivated members of communities that carry out critical ecosystem services such as carbon and nutrient cycling and bioremediation, in addition to secure and sustainable bioenergy production. The future is full of possibilities when omic strategies merge to define how cells work, and ABPP plays an important role in filling in the gaps of our knowledge relating to the organisms and pathways of interest.

## Abbreviations

ABP: activity-based probe
ABPP: activity-based protein profiling
GH: glycoside hydrolase
LC–MS: liquid chromatography–mass spectrometry
PTM: post-translational modification
